# Luteolin Attenuates Airway Mucus Overproduction via Inhibition of the GABAergic System

**DOI:** 10.1038/srep32756

**Published:** 2016-09-06

**Authors:** Mei-Lin Shen, Chen-Hung Wang, Ching-Huei Lin, Ning Zhou, Shung-Te Kao, Dong Chuan Wu

**Affiliations:** 1 Graduate Institute of Chinese Medicine, China Medical University, Taichung, Taiwan; 2 Graduate Institute of Clinical Medical Science, China Medical University, Taichung, Taiwan; 3 Translational Medicine Research Center, China Medical University Hospital, Taichung, Taiwan

## Abstract

Airway mucus overproduction is one of the most common symptoms of asthma that causes severe clinical outcomes in patients. Despite the effectiveness of general asthma therapies, specific treatments that prevent mucus overproduction in asthma patients remain lacking. Recent studies have found that activation of GABA_A_ receptors (GABA_A_R) is important for promoting mucus oversecretion in lung airway epithelia. Here, we report that luteolin, a natural flavonoid compound, suppresses mucus overproduction by functionally inhibiting the GABAergic system. This hypothesis was investigated by testing the effects of luteolin on goblet cell hyperplasia, excessive mucus secretion, and GABAergic transmission using histological and electrophysiological approaches. Our results showed that 10 mg/kg luteolin significantly decreased the number of goblet cells in the lung tissue and inhibited mucus overproduction in an *in vivo* asthma model induced by ovalbumin (OVA) in mice. Patch-clamp recordings showed that luteolin inhibited GABA_A_R-mediated currents in A549 cells. Furthermore, the inhibitory effects of luteolin on OVA-induced goblet cell hyperplasia and mucus overproduction were occluded by the GABA_A_R antagonist picrotoxin. In conclusion, our observations indicate that luteolin effectively attenuates mucus overproduction at least partially by inhibiting GABA_A_Rs, suggesting the potential for therapeutic administration of luteolin in the treatment of mucus overproduction in asthma patients.

Asthma is a major public health issue that affects as many as 300 million people worldwide (of all ages), with a global prevalence ranging from 1% to 21% in adults; up to 20% of children aged 6 to 7 years old experience severe wheezing episodes within a year^1^,^2^,^3^. Although current therapies have effectively decreased the rate of asthma hospitalizations and asthma-related deaths, a significant number of asthma patients do not respond well to existing therapies^4^,^5^,^6^.

Mucus overproduction is one of the most important pathological features of chronic inflammatory airway diseases that contribute to the morbidity and mortality associated with asthma, chronic obstructive pulmonary disease, and cystic fibrosis^7^,^8^,^9^. Mucus overproduction is characterized by enhanced mucin gene expression and mucin synthesis, excessive mucus secretion, goblet cell hyperplasia, and phlegm production^8^,^10^,^11^. Under normal conditions, mucus plays a protective role in the airway by preventing the entrance of invading pathogenic microorganisms, chemicals, and particles. However, excessive mucus secretion in the asthmatic airway reduces epithelial ciliary motility, damages local defensive function, promotes bacterial colonization, and increases recurrent infection^12^. More severely, airway obstruction because of mucus overproduction is one of the major causes of mortality in asthma patients. Mucin 5ac (Muc5ac) protein, encoded by the Muc5ac gene is the major type of mucin, which is the main biochemical component of mucus expressed in the airway epithelium in respiratory diseases^11^,^13^,^14^. Recent studies showed that chronic mucus hypersecretion increases the risk of lung cancers due to decreased carcinogen clearance^15^,^16^,^17^. Therefore, inhibition of mucus overproduction and mucus obstruction is a crucial therapeutic target for reducing serious airway complications.

The GABAergic system is the major inhibitory neurotransmitter system in the central nervous system (CNS), consisting of the neurotransmitter gamma-aminobutyric acid (GABA) and GABA receptors. A type GABA receptors (GABA_A_Rs), a class of pentameric ion channels highly permeable to chloride anions (Cl^−^), are the major form of ionotropic GABA receptors mediating inhibitory transmission in the brain and spinal cord^18^. Interestingly, recent studies showed that the GABAergic system, particularly GABA_A_Rs distributed in airway epithelial cells, is highly associated with mucus overproduction in respiratory diseases^19^,^20^. Allergic responses can cause the increased expression of both GABA_A_Rs and enzymes for GABA synthesis in airway epithelial cells. Activation of GABA_A_Rs causes depolarization of epithelial cells followed by Cl^−^ efflux from the cell. The transfer of Cl^−^ is proposed to create an osmotic gradient across the epithelium and lead to secretion of fluid into the airway lumen^21^. Furthermore, enhanced GABAergic signaling also results in epithelial proliferation and goblet cell hyperplasia^20^. Therefore, inhibition of GABA_A_R activation is considered a new strategy to relieve symptoms of mucus overproduction and attenuate the progression of respiratory diseases.

Flavonoids are a class of natural compounds containing two phenyl rings and a heterocyclic ring. Various flavonoids have been identified as positive, negative, or biphasic modulators of GABA_A_Rs^22^. However, it is difficult to infer from previous studies which compound directly targets airway GABA_A_Rs because GABA_A_Rs are assembled from a range of subunit isoforms and different flavonoids may have different or even opposite pharmacological behaviors depending on the subunit isoforms used. In the present study, we found that luteolin (3′,4′,5,7-tetrahydroxyflavone), a natural flavonoid found in *Perilla frutescens* and many other medical herbs^23^,^24^,^25^, directly inhibits GABA_A_R-mediated current responses in lung epithelial cells (A549). Furthermore, luteolin reduced goblet cell hyperplasia, mucin gene expression, and mucus accumulation in the airway in an *in vivo* model of mucus overproduction induced by ovalbumin (OVA), likely by inhibiting GABA_A_R activation. These data suggest the therapeutic potential of luteolin for treating mucus hypersecretion and related airway complications.

## Results

### Generation and characterization of the mucus overproduction model

**Figure 1:**
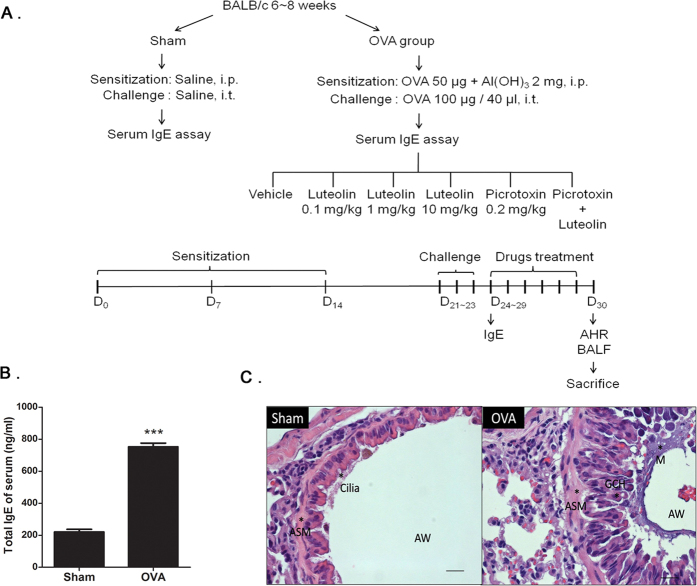
Experimental flow and verification of the OVA-induced mucus overproduction model. (**A**) Schematic flow chart of the experimental design. (**B**) Validation test of serum IgE assay using blood samples collected on day 24 in the sham (n = 40) and OVA groups (n = 112). The bars represent means ± S.E.M. Significance differences were determined by two-tailed, unpaired Student’s t-test, ***indicates *P* < 0.01. (**C**) Morphological changes of the lung from sham and OVA-treated mice. The sections were stained with hematoxylin and eosin (H&E) and analyzed with a microscope at a magnification of ×63. ASM, airway smooth muscle cells hypertrophy and hyperplasia; AW, airway; GCH, goblet cell hyperplasia; M, excess mucus secretion.

Mucus overproduction was studied in a murine allergic asthma model induced by OVA sensitization and challenge^26^ (Fig. 1A). To demonstrate that asthma responses were successfully induced, we examined serum IgE levels as an indication of asthma severity in all experimental animals after OVA challenges^27^,^28^,^29^,^30^. Our data showed that mice receiving OVA (n = 112) treatments exhibited significantly higher IgE levels than the sham group (n = 40, *P* < 0.001; Fig. 1B). Histological assessments also showed that lung sections from OVA-treated mice displayed epithelial alteration, epithelial shedding, mucus hypersecretion, ciliated cell loss, and goblet cell hyperplasia in the airway (Fig. 1C). These changes are consistent with clinical observations of mucus hypersecretion in asthma patients^8^. Together, these results revealed hallmarks of mucus overproduction in the OVA-induced asthma model.

### Luteolin reduced OVA-induced airway goblet cell hyperplasia and mucus occlusion

**Figure 2:**
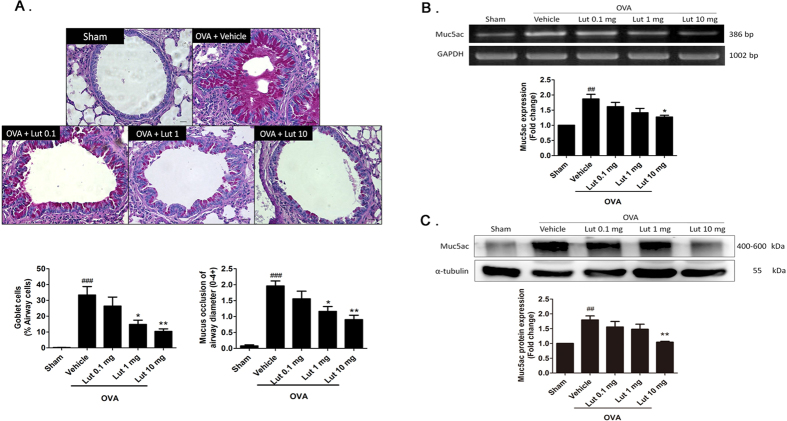
Effects of luteolin on OVA-induced goblet cell hyperplasia, mucus occlusion and Muc5ac mucin expression. (**A**) Representative lung tissue sections stained with PAS stain (×40 magnification) from the sham group or OVA-treated mice receiving vehicle or luteolin (0.1, 1, 10 mg/kg, i.p.). The number of goblet cells and mucus occlusion were significantly reduced in mice treated with 1 or 10 mg/kg (n = 9 and 8) but not with 0.1 mg/kg luteolin (n = 9), compared to mice receiving vehicles (n = 8). (**B**) Representative and quantitative data of Muc5ac gene expression assessed by RT-PCR (n = 7 for each group). (**C**) Representative and quantitative data of Muc5ac protein expression in each group by Western blot assay (n = 5 for each group). Full-length blots are presented in Supplementary Fig. 1A–D. The bars represent means ± S.E.M. Significant differences were determined by Kruskal-Wallis one-way ANOVA. ^##^*P* < 0.01 and ^###^*P* < 0.001 indicate significance differences compared to the sham group. ^*^P < 0.05 and ^**^P < 0.01 indicate significance differences compared to the OVA + vehicle group. Lut, luteolin.

We next tested whether luteolin can inhibit OVA-induced mucus overproduction. The periodic acid-Schiff (PAS) stain of airway sections showed that airway goblet cell hyperplasia and mucus overproduction were observed in OVA-treated mice but not in the sham group (Fig. 2A). In the OVA group, goblet cells have increased to 33.3 ± 5.3% of the total airway cells compared to the sham group (0.2 ± 0.13%, *P* < 0.001). Treatment with 1 mg/kg or 10 mg/kg luteolin significantly decreased goblet cell hyperplasia to 14.7 ± 2.7% and 10.3 ± 1.6%, respectively (*P* < 0.05 and *P* < 0.01 compared with the OVA group). The mean score of mucus occlusion of the airway diameter was 1.96 ± 0.15 in the OVA group, which was significantly higher than 0.07 ± 0.03 in the sham group (*P* < 0.001). With 1 mg/kg or 10 mg/kg luteolin treatment, the airway mucus occlusion scores significantly decreased to 1.16 ± 0.15 and 0.9 ± 0.14 (*P* < 0.05 and *P* < 0.01 compared to the OVA group), respectively.

The expression of Muc5ac mucin RNA and protein is a marker of goblet cell metaplasia in mouse airways^14^. Furthermore, Muc5ac is the main type of mucin in respiratory diseases with mucus hypersecretion, particularly in the asthmatic state^13^,^31^,^32^. The expression levels of Muc5ac mRNA increased significantly in the OVA group compared to the sham group (*P* < 0.05) in mouse lung tissue using reverse transcription polymerase chain reaction (RT-PCR) (Fig. 2B). When detected by Western blotting, Muc5ac protein produced bands with a high molecular mass of 400 to 600 kDa that mainly stayed near the gel entrance (Supplementary Fig. 1)^33^,^34^. To better separate bands of Muc5ac and the loading control alpha-tubulin, equal amount of samples were loaded onto 6% and 8% gels in parallel for the detection of Muc5ac and alpha-tubulin, respectively. The expression level of Muc5ac exhibited a significant increase, consistent with its mRNA levels in OVA-treated animals (Fig. 2C). We next examined the effect of luteolin on OVA-induced Muc5ac upregulation. Our results showed that treatment with luteolin (10 mg/kg) significantly attenuated the production of Muc5ac mRNA and protein (*P* < 0.05, Fig. 2B,C). Together, these results indicate a protective effect of luteolin on mucus overproduction in an *in vivo* asthma model.

### Luteolin inhibited airway inflammation and airway hyper-reactivity (AHR) in OVA-treated mice

**Figure 3:**
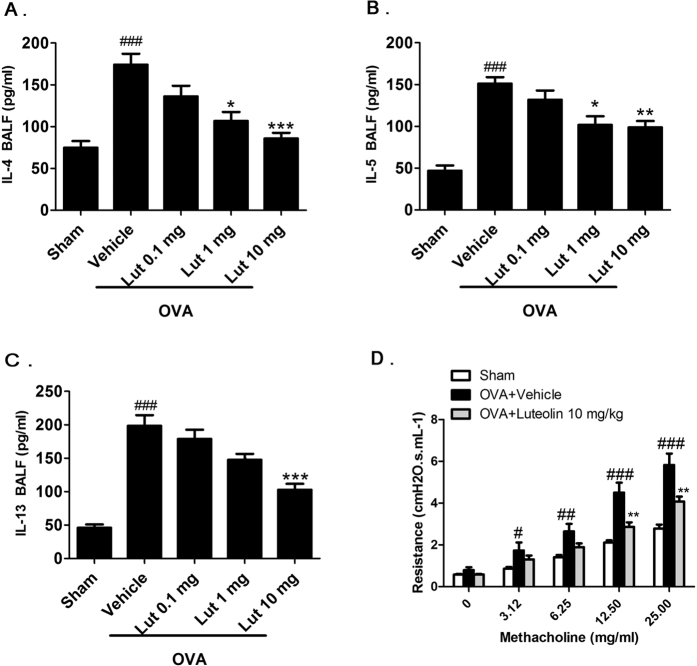
Effects of luteolin on OVA-induced airway inflammation and hyper-reactivity. (**A–C**) Expression levels of IL-4 (**A**), IL-5 (**B**), and IL-13 (**C**) in BALF were significantly higher in mice receiving OVA sensitization and challenge (n = 9) compared to mice receiving sham treatment (n = 6). In OVA-treated animals, BALF IL-4, IL-5 and IL-13 levels were dose-dependently inhibited by 0.1 mg/kg (n = 9), 1 mg/kg (n = 8), and 10 mg/kg luteolin (n = 8). (**D**) Pulmonary resistance in animals of the sham (n = 9), OVA+vehicle (n = 9), and OVA+10 mg/kg luteolin groups (n = 9) showed dose-dependent AHR in response to 3.125, 6.25, 12.5, and 25.0 mg/ml methacholine. The bars represent means ± S.E.M. Significance differences were determined by Kruskal-Wallis one-way ANOVA. ^#^*P* < 0.05, ^##^*P* < 0.01, and ^###^*P* < 0.001 indicate significance differences compared to the sham group. ^*^P < 0.05, ^**^P < 0.01, and ^***^P < 0.001 indicate significance differences compared to the OVA+vehicle group. Lut, luteolin.

The OVA sensitization and airway challenges induced significant inflammatory responses, which were demonstrated as significant upregulation of interleukin-4 (IL-4), IL-5, and IL-13 levels in the bronchoalveolar lavage fluid (BALF). Treatment of mice with luteolin (1 or 10 mg/kg, i.p.) produced a decreased pattern of IL-4, IL-5, and IL-13 expression (Fig. 3A–C). AHR was tested in sham and OVA-treated mice receiving vehicle or 10 mg/kg luteolin by measuring airway resistance in response to methacholine on day 30 after the first immunization of OVA. The OVA-treated animals receiving vehicle only showed enhanced airway resistance compared to the sham group. Luteolin treatments significantly suppressed airway resistance in response to 12.5 and 25.0 mg/ml methacholine (Fig. 3D). These results indicate that luteolin has a protective effect against airway inflammation and hyperresponsiveness in OVA-treated animals.

### Expression of GABA_A_ receptors in mouse lung tissue and A549 cells

**Figure 4:**
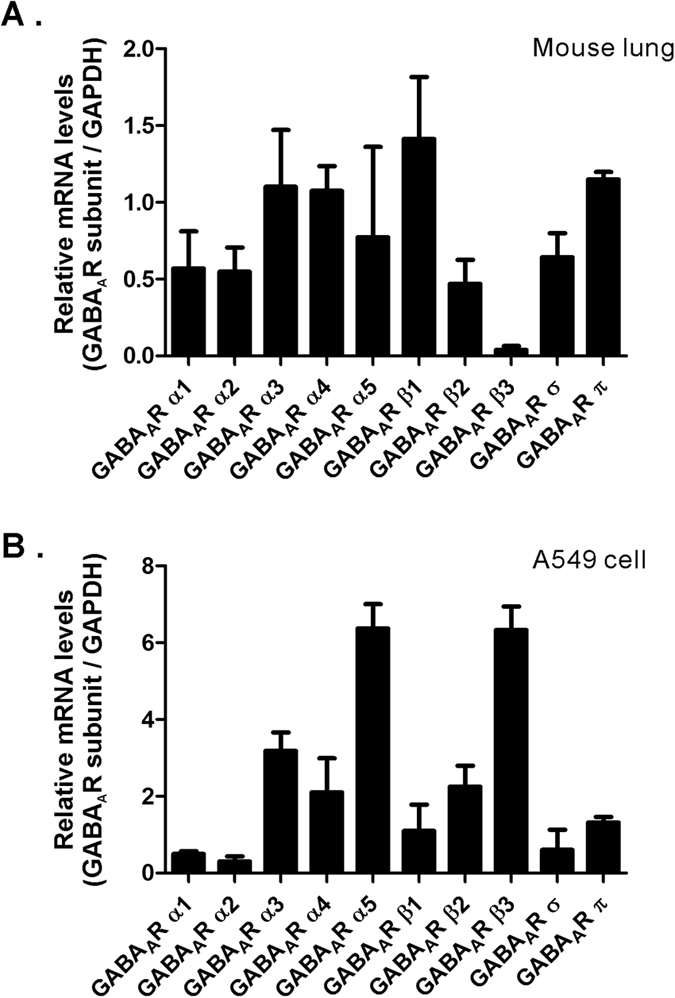
Expression of GABA_A_R subunits in mouse lung tissue and human alveolar epithelial A549 cells. (**A**) The normalized mRNA expression levels of murine GABA_A_R α1-5, β1-3, σ, and π subunits in mouse lung tissue. (**B**) The normalized mRNA expression levels of human GABA_A_R α1-5, β1-3, σ, and π subunits in A549 cells.

To determine whether luteolin reduces mucus overproduction by inhibiting GABA_A_Rs, we used an RT-PCR assay to investigate the expression of different GABA_A_R subunit genes in both mouse lung tissue and A549 cells, a human alveolar basal epithelial cell line. Consistent with previous studies^20^,^35^,^36^, we found that various types of GABA_A_R subunits are differentially distributed in naive mouse lung tissue and A549 cells (Fig. 4). These data confirm that GABA_A_Rs are expressed in the lung and may be a possible molecular target of luteolin.

### Luteolin reduced GABA_A_ receptor-mediated current responses in A549 cells

**Figure 5:**
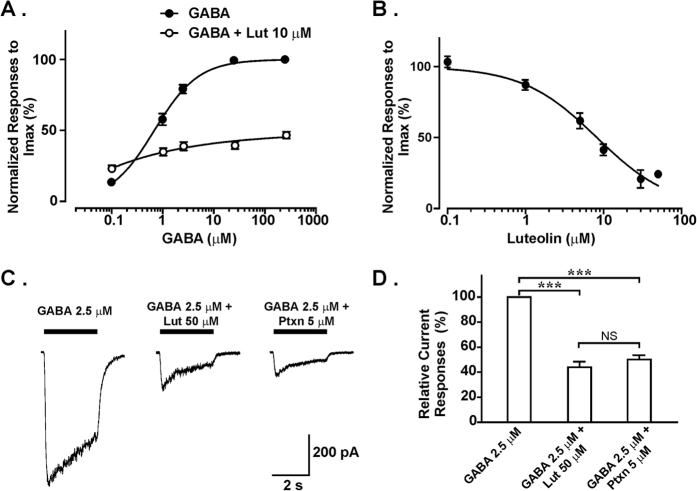
Modulatory effects of luteolin on GABA-induced currents in A549 cells. (**A**) Modulatory effects of luteolin (10 μM) on dose-response curves of GABA-induced currents in A549 cells (n = 12 to 18). (**B**) The dose-response curve of luteolin (0.1, 1, 5, 10 and 30 μM) blockade on GABA (2.5 μM)-mediated currents in A549 cells (n = 11). (**C**) Representative traces of GABA-induced currents in A549 cells and the inhibitory effects of luteolin or picrotoxin. (**D**) Summary of the effect of luteolin and picrotoxin on GABA-induced currents (n = 11). The bars represent means ± S.E.M. Significance differences were determined by Kruskal-Wallis one-way ANOVA. ^*^P < 0.05, ^**^P < 0.01, and NS indicates no significance. Lut, luteolin; Ptxn, picrotoxin.

To determine whether luteolin reduces mucus overproduction by direct inhibition of GABA_A_Rs in the asthma model^20^, we tested the modulatory effect of luteolin on GABA-induced current responses in A549 cells using patch-clamp recordings. Our data showed that GABA (0.1–250 μM) evoked whole-cell currents in A549 cells in a dose-dependent manner with an EC_50_ of 0.69 μM (Fig. 5A, solid circles). At 10 μM, luteolin inhibited GABA-mediated currents, with greater inhibitory effects at higher GABA concentrations (with the highest reduction of 60.3% with 25 μM of GABA, Fig. 5A, open circles). Treatment with different concentrations of luteolin inhibited GABA (2.5 μM)-induced currents in a dose-dependent manner, with an IC_50_ of 8.2 μM (Fig. 5B). These results suggest that luteolin directly targets GABA_A_Rs and inhibits their functions in human alveolar basal epithelial cells.

We next compared the inhibitory effects between luteolin and picrotoxin, a well-established GABA_A_R antagonist^18^, on GABA_A_R activation in the airway. When current responses were induced with 2.5 μM of GABA in A549 cells, 50 μM of luteolin decreased GABA_A_R currents to 44.0 ± 4.5% of the original responses (Fig. 5C,D). Such an effect was comparable to the inhibitory effect of 5 μM picrotoxin, which reduced GABA currents to 50.1 ± 3.4% in A549 cells under the same conditions (*P *> 0.05 between luteolin and picrotoxin). These results demonstrate that luteolin has a strong negative modulatory effect on GABA_A_R activation and further indicate that the effect of luteolin on mucus overproduction may be due to blockade of GABA_A_Rs.

### Effects of luteolin and picrotoxin on OVA-induced airway goblet cell hyperplasia and mucus occlusion *in vivo*

**Figure 6:**
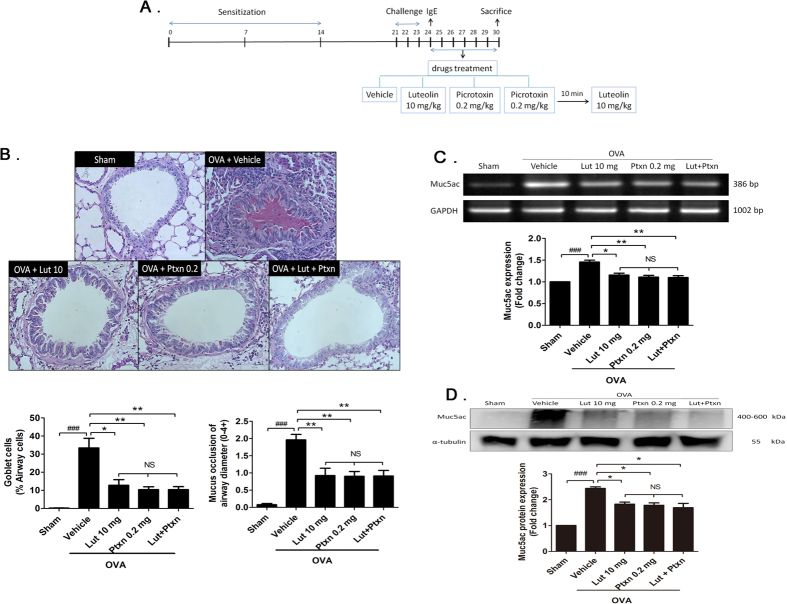
Luteolin decreases airway mucus expression by blocking GABAAR activation. (**A**) Schematic flow chart of the experimental design. (**B**) Representative PAS-staining (×40 magnification) of lung tissue sections from mice of the sham group (n = 8), OVA-sensitized and challenged mice treated with vehicle (n = 10), 0.2 mg/kg picrotoxin (n = 9), 10 mg/kg luteolin (n = 8), or with 10 mg/kg luteolin plus 0.2 mg/kg picrotoxin (n = 9). (**C**) Muc5ac gene expression was assayed by RT-PCR (n = 8 for each group). (**D**) Muc5ac protein expression assessed by Weston blot assay (n = 6 for each group). Full-length blots are presented in Supplementary Figure 1E–H. The bars represent means ± S.E.M. Significance differences were determined by Kruskal-Wallis one-way ANOVA. ^###^*P* < 0.001 indicates a significance difference compared to the sham group. ^*^P < 0.05 and ^**^P < 0.01 indicate significance differences compared to the OVA + vehicle group. NS indicates no significance. Lut, luteolin; Ptxn, picrotoxin.

To further investigate the pharmacological mechanism of luteolin on mucus overproduction, we blocked GABA_A_R activation using the selective GABA_A_R antagonist picrotoxin and then tested whether luteolin continues to have an effect on mucus overproduction. To test this hypothesis, we treated OVA-sensitized and -challenged mice with the following: (1) picrotoxin (0.2 mg/kg), (2) luteolin (10 mg/kg), or (3) combined treatments of picrotoxin (0.2 mg/kg) and luteolin (10 mg/kg) (Fig. 6A). According to histological assessments, goblet cell hyperplasia and mucus occlusion induced by OVA were significantly reduced by luteolin (12.77 ± 3.10%, n = 8) and picrotoxin (10.33 ± 1.62%, n = 9) alone to a similar extent (*P *> 0.05, Fig. 6B). However, the combined treatment of luteolin and picrotoxin did not yield significantly better effects (*P *> 0.05 compared to luteolin alone or picrotoxin alone). Furthermore, the expression levels of Muc5ac mRNA and protein were also examined in animals receiving these treatments. Luteolin or picrotoxin alone decreased OVA-induced Muc5ac upregulation to a similar extent, and Muc5ac levels were not further decreased by combined treatment with both drugs (*P *> 0.05, Fig. 6C,D). Our results clearly show that luteolin did not produce any further effect after GABA_A_Rs were blocked by picrotoxin. Similarly, picrotoxin did not further extend the effect of luteolin, indicating that these two drugs target the same pathway to exert their anti-asthmatic effects by blocking GABA_A_R activation. Taken together with our electrophysiological data, these results strongly indicate that luteolin effectively attenuates mucus overproduction in the asthma model by inhibiting GABAergic signaling pathways.

## Discussion

Luteolin has been predicted to exert its anti-asthmatic effects by acting as an anti-inflammatory drug^25^,^37^,^38^,^39^. However, we found that luteolin strongly inhibited GABA_A_R-mediated responses in lung epithelia cells. Moreover, the GABA_A_R antagonist picrotoxin occluded the effect of luteolin on goblet cell hyperplasia and mucus overproduction, suggesting a GABA_A_R-dependent mechanism. Our findings indicate that some flavonoid chemicals may exert their protective effect on mucus overproduction not only via an anti-inflammatory effect but also through inhibition of GABAergic signaling.

### Distribution of GABA signaling in the airway

Many studies showed that GABA synthetic enzymes and GABA_A_Rs are expressed in the respiratory system and alveolar cell lines. The glutamic acid decarboxylase (GAD65/67) is expressed in mouse pulmonary neuroendocrine cells^40^, mouse alveolar epithelial type II cells^41^, monkey bronchial epithelial cells^42^, A549 cells^20^, and human airway epithilum^43^,^44^. Previous studies and our present findings both demonstrated the expression of mRNA or proteins of various GABA_A_R subunits, including α1-3, α5, β2, β3, γ1, γ3, ε, π, and σ, in the human lung epithelia cell line (A549), human lung epithelia, and mouse lung tissue^20^. We found that α3, α4, and β1 subunits are highly expressed in mouse lung tissue, whereas α3, α5, β2, β3, and π are the most abundant subunits in A549 cells^20^,^36^. Normally, the αβ composition is required to form a functional GABA_A_R, whereas αβγ2 is the major form of brain GABA_A_R responsible for inhibitory synaptic transmission. Interestingly, a major brain GABA_A_R subunit, γ2, is not likely to be present in the respiratory system^35^, which indicates that differences in the composition of functional GABA_A_Rs exist between those of the airway and the CNS. Furthermore, our electrophysiological results found that GABA induced large current responses in A549 cells with an EC_50_ of 0.69 μM, which is within the same range reported by other groups (EC_50_ of 2.45 μM)^36^. Because subunits, including α1-3, 5, β2, β3, γ1, 3, ε, and π are found in human airway epithelium^42^, luteolin also targets GABA_A_Rs in the human airway.

### Mucus production and the GABAergic signaling

Recent studies indicated that the GABAergic signaling system is tightly associated with mucus accumulation in the airway in an animal asthma model. Nicotine treatment induced GAD and GABA_A_R expression and contributes to nicotine-induced overproduction of mucin *in vitro* and *in vivo*^42^. In the mouse second-hand cigarette smoke model, mucous cell metaplasia induced by cigarette smoke/nicotine requires GABA_A_R upregulation^45^. In the human airway epithelium of cigarette smokers, expression of the GABAergic system has been detected, and GAD67 and Muc5ac overproduction are associated with smoking^44^. The enhanced autocrine GABA signaling results in chloride efflux, depolarization, cell proliferation, and mucus overproduction of airway epithelia cells^20^,^36^. Together with these previous studies^20^, our study demonstrates that the GABA_A_R inhibitor picrotoxin prevents allergen-induced goblet cell transformation and mucus overproduction. These results indicate that the GABAergic system is a potential therapeutic target for reducing mucus overproduction.

### Pharmacological effects of luteolin

As a natural flavonoid, luteolin has multiple biological activities, such as anti-inflammatory, antioxidant, anti-carcinogenic, and anti-allergic effects^25^,^38^. However, studies that used luteolin as a GABA_A_R modulator to treat asthmatic responses remain lacking. Some types of flavonoids have positive, negative, or neutralizing modulatory effects on GABA_A_Rs^22^. Here, we found that luteolin potentiated very low dose GABA (0.1 μM)-induced currents in A549 cells (Fig. 3). However, when current responses were induced by higher doses (2.5 μM GABA) in A549 cells, luteolin showed an inhibitory effect in a dose-dependent manner. The inhibitory effect of 50 μM luteolin on GABA currents was comparable to that of 5 μM picrotoxin, a well-established GABA_A_R antagonist. Furthermore, the effect of luteolin on mucus overproduction and goblet cell hyperplasia was not further extended by picrotoxin, suggesting that luteolin exhibits its effect through inhibition of GABA_A_R activation, thereby occluding the effect of picrotoxin.

Our results are consistent with previous reports on the protective effects of luteolin against mucus secretion *in vitro* and asthmatic responses *in vivo*. For example, luteolin reduces Muc5ac expression stimulated by epidermal growth factor or phorbol 12-myristate 13-acetate in NCI-H292 cells^37^. Luteolin inhibits bleomycin-induced lung fibrosis^46^ and alleviates OVA-induced bronchoconstriction and airway hyperreactivity^39^. Consistent with previous studies, we also found that luteolin suppressed OVA-induced IL upregulation and AHR, suggesting that luteolin may be used as an anti-asthmatic agent. Our results confirm that luteolin has strong inhibitory effects on mucus overproduction in the *in vivo* asthma model induced by OVA and advances current understanding of the effect of luteolin on the GABA_A_R signaling pathway.

### Safety of luteolin as a GABA_A_R modulator

GABA_A_R antagonists are generally considered a group of convulsants. Systematic administration of GABA_A_R antagonists that cross the blood-brain barrier typically causes seizure activities and other side effects in the CNS. However, luteolin is generally considered safe^47^,^48^ because it is contained in many edible plants, such as broccoli, bird chili, and onion leaves^49^. We showed that luteolin acts as an allosteric modulator of GABA_A_Rs. After systemic administration of luteolin, we did not observe convulsive or other abnormal behaviors in animals during our study. This may be due to the limited permeability of luteolin through the blood brain barrier or its different modulatory effects on brain GABA_A_Rs. These possibilities must be tested.

## Conclusion

Our observations indicate that luteolin effectively attenuates mucus overproduction and goblet cell hyperplasia in an animal asthma model at least partially by inhibiting GABA_A_R activities. These data suggest the potential for therapeutic administration of luteolin in the treatment of mucus overproduction in asthma patients.

## Methods

### Chemicals and Reagents

Luteolin, picrotoxin, ovalbumin and the PAS staining kit and other chemical reagents were purchased from Sigma (Sigma-Aldrich, St. Louis, MO, USA). Anti-Muc5ac and anti-alpha-tubulin antibodies were purchased from Santa Cruz Biotechnology (sc-21701, sc-12462-R, Dallas, TX, USA). Horseradish peroxidase (HRP)-conjugated secondary antibodies were purchased from Pierce Biotechnology (Thermo Fisher Scientific, Rockford, IL, USA). IL-4, IL-5 and IgE ELISA kits were purchased from BD Biosciences (San Diego, CA, USA). The IL-13 kit was purchased from R&D Systems (Minneapolis, MN, USA). OVA for intraperitoneal (i.p.) injection was adsorbed to aluminum hydroxide adjuvant (Santa Cruz, Dallas, TX, USA) at a ratio of 50 μg to 2 mg in 200 μl PBS. OVA for intratracheal (i.t.) injection was dissolved in saline at a final concentration of 2.5 mg/ml. Luteolin (Sigma-Aldrich, St. Louis, MO, USA) was prepared in DMSO and diluted with saline. Picrotoxin (Sigma-Aldrich, St. Louis, MO, USA) was prepared in DMSO and diluted with saline. Saline with 0.5% DMSO was used as a vehicle in control groups. The final concentration of DMSO in all reaction mixtures was less than 0.5%.

### Animals

Adult male BALB/c mice, weighing 18–22 g, were obtained commercially from BioLasco co., Ltd. (Taipei, Taiwan). Animals were housed under controlled laboratory conditions with a 12-h dark-light cycle. The experiments that involved experimental animals were carried out in accordance to the Institutional Guidelines of the China Medical University for the Care and Use of Experimental Animals (IGCMU-CUEA) and were approved by the Institutional Animal Care and Use Committee (IACUC) of China Medical University (Taichung, Taiwan).

### Murine model of asthma

The asthma model and validation were established according to a previously described protocol^20^,^30^,^50^. As shown in Fig. 1, mice were administered an i.p. injection of 50 μg of ovalbumin (OVA, adsorbed in 2 mg aluminum hydroxide in 200 μl PBS) on days 0, 7, and 14, followed by challenge of i.t. instillation of OVA (100 μg in 40 μl of saline) on days 21, 22, and 23. Mice of the OVA group were randomly allocated to six groups, receiving treatments of 0.1 mg/kg/day luteolin, 1 mg/kg/day luteolin, 10 mg/kg/day luteolin, 0.2 mg/kg/day picrotoxin, combined treatment of luteolin plus picrotoxin (10 and 0.2 mg/kg/day, respectively), or vehicle by i.p. injection once daily for 6 days. The mice were sacrificed on the next day after the last drug administration, and airway tissues were collected for further analysis.

### Estimation of serum IgE using ELISA assay

Blood samples were drawn from the orbital sinus and collected on day 24 in the sham (n = 40) and the OVA group (n = 112). The serum IgE level was measured using an enzyme-linked immunosorbent assay antibody and ELISAs were performed according to the manufacturer’s instructions.

### BALF Collection and Cytokine ELISA assay

Mice were anesthetized, and the trachea was cannulated while the thorax was gently massaged. Lungs were lavaged twice with 1 ml of saline. The covered lavage samples were cooled on ice and centrifuged at 1200 rpm for 10 min at 4 °C. The supernatant was collected and stored at −80 °C for ELISA assays. IL-4, IL-5 and IL-13 levels in BALF were measured using specific mouse IL-4, IL-5 and IL-13 ELISA kits and ELISAs were performed according to the manufacturer’s instructions.

### Histopathological analysis

The left lung tissue was collected and fixed with 4% paraformaldehyde (wt/vol) in PBS at 4 °C for 24 h. The tissues were embedded in paraffin and cut into 2–5 μm sections. The slices were dewaxed by immersion in xylene, rehydrated in descending concentrations of an ethanol series (100% to 70%), and stained with hematoxylin and eosin (H&E). All slides were observed for pathological changes under light microscopy. Goblet cell hyperplasia and mucus occlusion were assayed with a PAS staining kit as previously described^30^,^51^. Slides were visualized using an upright light microscope (Zeiss, Oberkochen, Germany) with bright field illumination. Images with 40x or 63x magnification were acquired with Zeiss Plan-Apochromat 40x/0.95 and 63x/1.4 objectives, respectively. Images were captured with an AxioCam HRm camera and processed using the Axiovision LE Rel4.4 software (Zeiss, Oberkochen, Germany). The number of goblet cells was determined as the percentage of total airway epithelial cells in each airway examined. The cross-sectional area of the airway lumen and mucus plugging was outlined by the “freehand selections” function of ImageJ and was automatically calculated by the same software. The grade of mucus plugging of the airways was assessed as percent occlusion of airway lumen on the following scale: 0, no mucus; 1, <10% occlusion; 2, 30% occlusion; 3, <50% occlusion; and 4, greater than approximately 80% occlusion.

### RNA isolation and reverse transcription polymerase chain reaction (RT-PCR)

Total RNA was isolated from murine lung tissue or cells using TRIzol reagent (Invitrogen Life Technologies, Carlsbad, CA, USA), according to the manufacturer’s instructions. Briefly, the RNA precipitate was washed twice by gentle vortexing with 70% ethanol, collected by centrifugation at 12000 rpm, dried under a vacuum for 5–10 min, dissolved in 200 μl RNase-free water (Promega, Madison, WI, USA), and incubated for 10–15 min at 55–60 °C. The RNA was quantified and assessed for purity by spectrophotometry at a wavelength of 260 nm. The integrity was assessed by 2% agarose gel electrophoresis and the RNA was visualized by ethidium bromide staining. The GABA_A_R subtype and Muc5ac mRNA transcripts were measured by RT-PCR as previously described^30^,^52^. Glyceraldehyde 3-phosphate dehydrogenase (GAPDH) was used as the endogenous control gene. The PCR primers for mice and humans were designed according to the published cDNA sequences and synthesized by MDBio Inc (Taipei, Taiwan). The primers sequences are listed in Table 1. Mouse or human GAPDH mRNA was used as the internal control in all experiments. The mRNA expression levels of Muc5ac and GABA_A_R subunits were measured by band intensities using the Gel Analysis method in ImageJ 1.49 and were normalized to the level of GAPDH.

### Protein extraction and Western blotting

The lung tissues were harvested and homogenized in RIPA lysis buffer containing proteinase inhibitors. After vortexing and a 10 min incubation on ice, the extract was centrifuged at 12000 rpm for 40 min at 4 °C. The supernatant protein extracts were collected and stored at −80 °C for future use. The protein concentration in the tissue extract was determined using a Bio-Rad protein assay kit.

For the detection of Muc5ac and alpha-tubulin, equal amounts of total protein sample (100 μg) were separated on 6% and 8% sodium dodecyl sulfate polyacrylamide gels, respectively, and transferred onto polyvinylidene difluoride (PVDF) membranes (Millipore, Bedford, MA, USA). To block nonspecific binding, the membranes were incubated for 1 h in blocking buffer (5% fat-free milk, 0.1 M Tris-HCl, 0.9% NaCl, 1% Tween-20, pH 7.4) at room temperature, and then incubated with anti-Muc5ac antibodies (1:1000) and alpha-tubulin antibodies (1:2000) overnight at 4 °C. Subsequently, membranes were incubated with an HRP-linked secondary antibody. The expression of proteins was detected using enhanced chemiluminescent (ECL) reagents (Millipore, Billerica, USA), and imaged on an ImageQuant LAS 4000 system (GE Healthcare, Buckinghamshire, UK). The protein levels of Muc5ac or alpha-tubulin were analyzed using Image J 1.49 software.

### Invasive Measurements of Airway AHR

Airway resistance was assessed as an increase in pulmonary resistance in response to increasing concentrations of aerosolized methacholine in anesthetized mice. Briefly, mice were anesthetized with 70–90 mg/kg pentobarbital sodium. The trachea was exposed and cannulated with an 18-gauge tracheal tube. A suture around the trachea was then tied to prevent air leak. Mice were mechanically ventilated using a computer-controlled small animal ventilator (flexiVent; Scireq, Montreal, Canada) with the following parameters: respiratory rate of 150 breaths/min, tidal volume of 10 ml/kg, inhalation: exhaustion ratio of 2:3, and positive end-expiratory pressure of 2–3 cm H_2_O. Aerosolized PBS and increasing concentrations of methacholine (3.125, 6.25, 12.5, and 25.0 mg/ml) were delivered to the animal and readings were recorded every 4 min for 12 sec at each concentration. Pulmonary resistance was calculated using the flexiWare software.

### Cell culture

The human alveolar basal epithelial cell line A549 was purchased from the American Type Culture Collection (A549, ATCC, Manassas, VA, USA). Cells were used at passage number 20–30 and maintained in Ham’s F12K medium with 10% fetal calf serum, 100 units/ml penicillin, and 100 mg/ml streptomycin at 37 °C in a 5% CO_2_ incubator with humidified atmosphere. All experiments were performed when cells were 80–90% confluent. For electrophysiological recording experiments, cells were seeded on poly-D-lysine-coated 12 mm glass coverslips in 24-well plates at a density of 5*10^4^ cells per well.

### Electrophysiology

The electrophysiology study was performed on A549 cells by examining GABA_A_R- mediated currents with whole-cell patch-clamp recordings^53^. Briefly, after removing the culture medium, cells were immersed in a solution containing (in mmol/l) 140 NaCl, 5.4 KCl, 10 HEPES, 1.0 MgCl_2_, 1.3 CaCl_2_, and 20 glucose (pH 7.4, 305–315 mOsm). Whole-cell recordings were performed under voltage-clamp mode using an Axopatch 200B (Molecular Devices, Sunnyvale, CA, USA). Whole-cell currents were recorded at a holding potential of −60 mV, and signals were low-pass filtered at 2 kHz and digitized at 10 kHz. GABA-mediated currents were evoked by a computer-controlled multibarrel fast perfusion system (Warner Instruments, Hamden, CT, USA). Maximum currents (I_max_) were evoked by the highest concentration of GABA as determined by the dose-response curves. Modulatory effects of luteolin or picrotoxin on GABA_A_Rs were tested on 2.5 μM GABA-induced currents on the same cell with and without 50 μM luteolin or 5 μM picrotoxin in the extracellular solution.

### Statistical analysis

All results are presented as the mean ± standard error of mean (S.E.M.). Statistical analysis was performed using the Prism statistical analysis program (GraphPad 6.0). Kruskal-Wallis one-way ANOVA test was used for statistical comparisons of 3 or more groups. Two-tailed unpaired Student’s t-test was used for statistical comparisons of two groups. *P* < 0.05 was considered significant for all tests. Statistical significance is presented as *P < 0.05; **P < 0.01; and ***P < 0.001.

## Additional Information

**How to cite this article**: Shen, M.-L. *et al*. Luteolin Attenuates Airway Mucus Overproduction via Inhibition of the GABAergic System. *Sci. Rep*. **6**, 32756; doi: 10.1038/srep32756 (2016).

## Supplementary Material

Supplementary Information

## Figures and Tables

**Table 1:** Primer sequences for GABA_A_R subunits, Muc5ac and GAPDH of mice and humans.

GENE (mouse)	Size (bp)	Sequence (5′-* *> 3′)	Tm (°C)	Accession number
GABAARα1	395	TATGGACAGCCCTCCCAAGA	60	NM_010250.4
		ATGGTGTACAGCAGAGTGCC		
GABAARα2	287	CCCGATGGCTCCAGGTTAAA	60	NM_008066.3
		GGGAGGGAATTTCGAGCACT		
GABAARα3	230	GCCAAGGGGAATCAAGACGA	60	NM_008067.4
		AGGGCCAAAACTGGTCACAT		
GABAARα4	399	TTGGGCCCGTTTCTGATGTT	60	NM_010251.2
		TTGGCACCTCCACTGACTTC		
GABAARα5	371	CCTGGGCTGGGAGAGCGAATC	56	NM_176942.4
		AGGGGACAGGCATGGGCATC		
GABAARβ1	246	CCATGACCACCATCAGCACT	60	NM_008069.4
		TATTGCCGTGGGCATCAACT		
GABAARβ2	408	TGGACCTAAGGCGGTATCCA	60	NM_008070.3
		TAGGGAGAGTTTCCCGGAGG		
GABAARβ3	341	GTTCCCACTCAGGTTAGGCT	60	NM_001038701.1
		GGATCCCAGAATAGGCGAGC		
GABAAR δ	301	AAGCTTATCCGCCTACAGCC	60	NM_008072.2
		GGAAGTGTAAGCTGAGCCGT		
GABAAR π	246	GGCTGAGAGGGAATGACTCG	60	NM_146017
		GGATCCCAGAATAGGCGAGC		
GAPDH	263	CCCTTAAGAGGGATGCTGCC	61	XM_011241212.1
		ACTGTGCCGTTGAATTTGCC		
GAPDH	1002	ATGGTGAAGGTCGGTGTGAAC	60	XM_001476707.3
		TTACTCCTTGGAGGCCATGTA		
Muc5ac	386	AGCTACAGTGCAACTGGACC	60	NM_010844.1
		GGACACAGATGATGGTGACA		
**GENE (human)**	**Size (bp)**	**Sequence (5′-***** *****> 3′)**	**Tm (°C)**	**Accession number**
GABAARα1	517	CATGCCCAACAAACTCCTGC	60	NM_000806
		AGCTGTTGCATAAGCCACCT		
GABAARα2	573	TGCTCCTGATGGCTCTAGGT	58	NM_000807
		TTCTTGTTGGGTTCTGGCGT		
GABAARα3	565	GGCTTGGAGATGCAGTGACT	60	NM_000808
		CGCTTGAGATGGAAGTGGGT		
GABAARα4	579	GCGGAGTGTCCCATGAGATT	60	NM_000809
		AACTTCCTGAGGGGGATTTG		
GABAARα5	535	TACGACAACAGACTTCGGCC	60	NM_000810
		TGATGTTCTCAGTGCCCACC		
GABAARβ1	513	TTTGTGCATGGGGTCACAGT	60	NM_000812
		GATCTTTGGCAGGGTCTCCC		
GABAARβ2	405	ACCATCCTCTCCTGGGTCTC	60	NM_021911
		GCTTCTGGGGTCTCCAAGTC		
GABAARβ3	554	GCTCGCCTATTCTGGGATCC	60	NM_000814
		TGATCCCGAGGGCAACTCTA		
GABAAR δ	552	ATGCTGGACCTGGACTAGCTA	64	NM_000815
		GAGGACAATGGCGTTCCTCA		
GABAAR π	594	CTCTTCTCCAATGGCACGGT	60	NM_014211
		ATCTTTGGCTGCCATCTGCT		
GAPDH	647	GAAAGCCTGCCGGTGACTAA	64	NM_001256799.2
		GATGGCATGGACTGTGGTCA		
