# Supplementary material for: Luteolin Attenuates Airway Mucus Overproduction via Inhibition of the GABAergic System
Source: Sci Rep. 2016 Sep 6;6:32756. doi: 10.1038/srep32756 (PMC5011760; doi:10.1038/srep32756)
Supplement: Supplementary Information [file srep32756-s1.pdf]

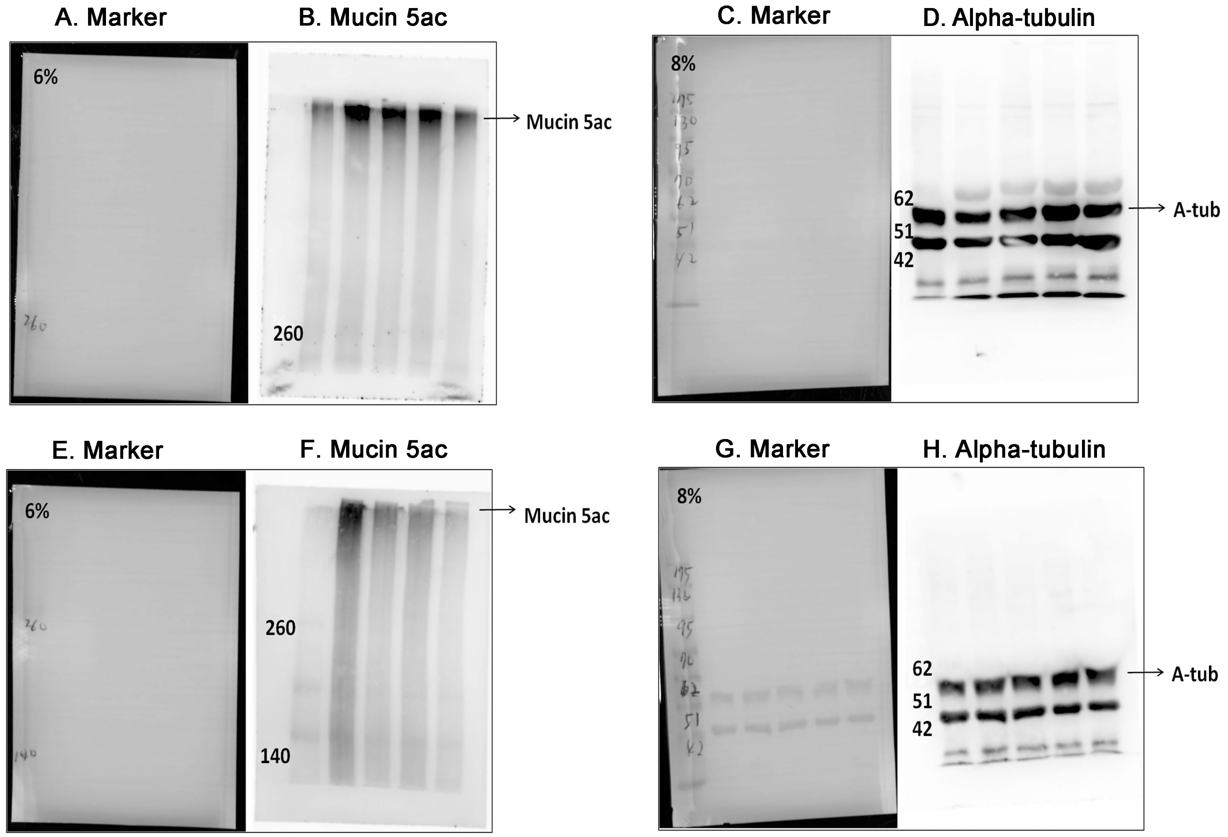

Supplementary Figure 1. Full-length images of the Western blots. A-D, Full-length blots of Figure 2C. E-H, Full-length blots of Figure 6D.
